# Supplemental Anti Vegf A-Therapy Prevents Rebound Neovascularisation After Fine Needle Diathermy Treatment to Regress Pathological Corneal (LYMPH)Angiogenesis

**DOI:** 10.1038/s41598-020-60705-z

**Published:** 2020-03-03

**Authors:** Viet Nhat Hung Le, Yanhong Hou, Felix Bock, Claus Cursiefen

**Affiliations:** 1 Department of Ophthalmology, Faculty of Medicine and University Hospital Cologne, University of Cologne, Köln, Germany; 2 Department of Ophthalmology, Hue College of Medicine and Pharmacy, Hue University, Hue, Vietnam; 3 Centre for Molecular Medicine Cologne (CMMC), University of Cologne, Köln, Germany

**Keywords:** Preclinical research, Preclinical research, Acute inflammation, Acute inflammation

## Abstract

Fine needle diathermy (FND) is an effective method to destroy and regress pathologic corneal blood and lymphatic vessels. However, it is unknown whether FND itself causes a rebound corneal neovascularisation and whether that can be prevented by VEGF blockade. In female BALB/c mice, the suture-induced inflammatory corneal neovascularisation model was used to induce hem- and lymphangiogenesis. Thereafter, prevascularized mice were divided into 2 groups: the combination therapy group received FND cauterization and subsequent VEGF TrapR_1_R_2_ eye drops three times per day whereas the monotherapy group was treated only with FND. Three, 7 and 14 days after the treatment, corneas were collected and stained with FITC-conjugated CD31 and LYVE-1 followed by Cy3-conjugated secondary antibody to quantify corneal blood and lymphatic vessels. Relative mRNA expression of VEGF in the cornea was quantified by using qPCR. FND cauterization as monotherapy significantly obliterated (lymph)angiogenesis at early time points; however, this treatment led to secondary corneal hem- and lymphangiogenesis associated with significant upregulation of pro(lymph)angiogenic VEGF-A, VEGF-C, VEGF-D and infiltration of macrophages. Combining FND cauterization with VEGF TrapR_1_R_2_ treatment prevented the undesired effect of the FND procedure alone and significantly better regressed corneal blood and lymphatic vessels at 1 week after the treatment compared to monotherapy and control group (p < 0.01).

## Introduction

The cornea is one of the few human tissues devoid of blood vessels (BVs) and lymphatic vessels (LVs). Therefore, it is an ideal location to investigate the mechanism of pathological hemangiogenesis and lymphangiogenesis. The avascular status of the cornea, also termed”corneal (lymph)angiogenic privilege”, is maintained by the balance between proangiogenic factors and anti-angiogenic factors^1,2^. Various diseases, however, can result in corneal neovascularisation (CoNV), i.e. sprouting of new vessels from the limbal vascular arcade into the cornea^3^. The presence of blood and lymphatic vessels disrupts not only the”angiogenic privilege” but also the ‘’immune privilege” of the cornea^1,4–6^ and leads to a significant increase of graft rejection after subsequent corneal transplantation^5–9^.

To regress pathological vessels prior to transplantation, different approaches have been used such as antiangiogenic argon laser or yellow dye laser and antisense oligonucleotide GS101^10–13^. More recently, photodynamic therapy and UV light crosslinking were reported to significantly regress corneal vessels in mouse models^14,15^. Another approach is anti-vascular endothelial growth factor (VEGF) therapy which has been broadly used off-label to inhibit progressive corneal angiogenesis and lymphangiogenesis^16,17^. However, anti-VEGFs as a monotherapy seems to be less effective in regressing mature vessels due to the fact that these vessels depend less on angiogenic growth factors^18^.

Fine needle diathermy (FND) is clinically used since 2000 to regress CoNV and is currently a promising clinical choice for managing mature pathologic corneal blood vessels^19,20^. The efficacy of this technique was documented by several studies in both clinical and animal settings^20–25^. We could recently show that the combination of FND and corticosteroids can regress both blood vessels and clinically invisible lymphatic vessels^25^. Based on clinical experience, many authors assume that FND in itself can induce rebound neovascularisation and should therefore be combined with anti-inflammatory eye drops or subconjunctival injection of anti-VEGF or with corneal angiography to lessen undesired effects of FND^19,20,23,24,26–28^.

However, so far there has been no formal evidence regarding expression level of angiogenic growth factor VEGF-A, VEGF-C and VEGF-D after FND treatment and FND induced rebound neovascularisation. Therefore, in this study, we investigated the potential additional angiogenic stimulus of FND procedure itself as a monotherapy and the effect of combined treatment of VEGF TrapR_1_R_2_ and FND on dampening the undesired effect of monotherapy as well as on regressing mature blood and lymphatic vessels and prevention of their recurrence.

## Results

### FND monotherapy induces secondary corneal hem- and lymphangiogenesis and this rebound neovascularisation can be rescued by combined supplemental VEGF-A blockade

**Figure 1:**
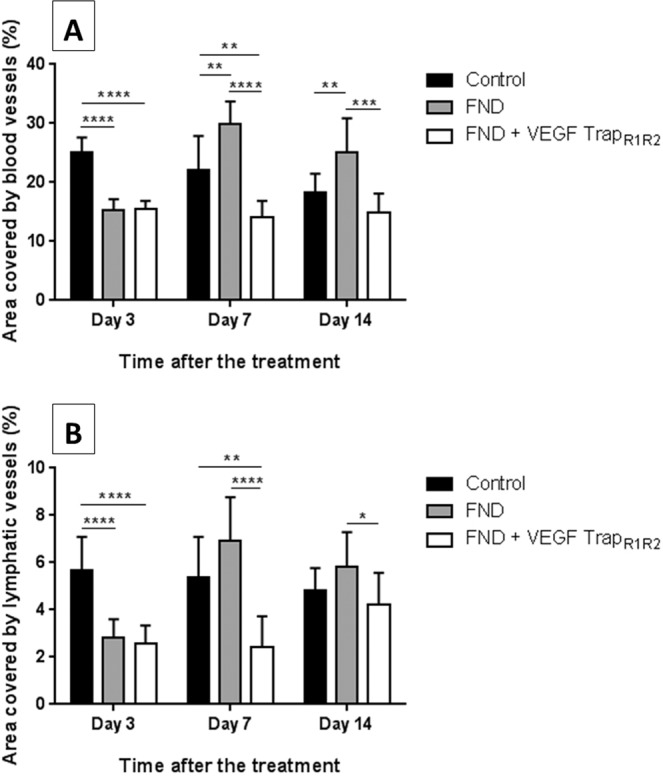
Rebound effect of FND monotherapy and preventive effect of supplemental anti-VEGF A blockade in combination with FND on regression the regrowth of blood vessels (**A**) and lymphatic vessels (**B**) after the treatment. FND monotherapy significantly obliterated both blood and lymphatic vessels at day 3; however, thereafter blood vessels significantly increased at day 7 in the monotherapy group compared to control group (p < 0.01). This undesired effect was rescued by VEGF-A blockade with significant reduction of both blood and lymphatic vessels in the combination therapy group compared to monotherapy group. n = 10 each group.

**Figure 2:**
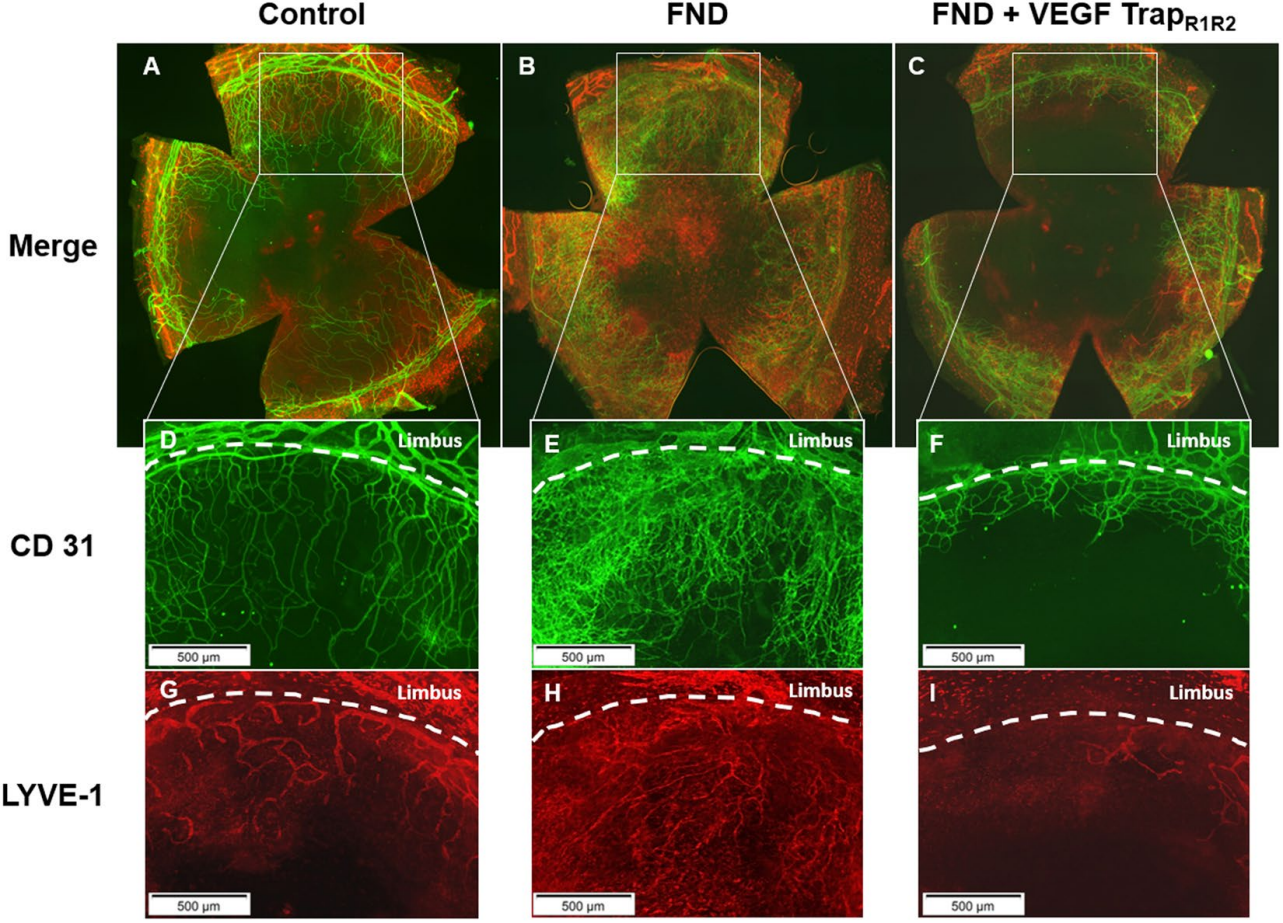
Corneal whole mounts at seven days after the FND treatment. Harvested corneas were stained with FITC-conjugated CD31 for blood vessels (**D–F**) and LYVE-1 followed by Cy3-conjugated secondary antibody for lymphatic vessels (**G–I**). (**A–C**) blood and lymphatic vessels merged. Immunohistochemistry pictures at day seven show the significant reduction of mature blood and lymphatic vessels seen with combination therapy as compared to FND monotherapy and without therapy.

To investigate whether FND as a monotherapy has a proangiogenic rebound effect, 14-day sutured corneas were treated by FND and then harvested subsequently three, 7 and 14 days after the FND treatment. Quantitative analysis of vascularized corneal area revealed that FND monotherapy is effective in regression of mature blood and lymphatic vessels at day 3; however, blood vessels were significantly increased in the monotherapy group (FND) compared to the control groups at day 7 and 14 (p < 0.01). Furthermore, we could show that the additional treatment with VEGF TrapR_1_R_2_ could prevent this rebound effect with a regression of blood vascularized (BV) area by 53% and lymphatic vessels (LV) by 65% compared to FND group (p < 0.0001) and 36% BV area and 55% LV area compared to control group (p < 0.01) at day 7 (Figs. 1, 2). This shows FND monotherapy to induce a VEGF-A dependent secondary neovascularisation.

### FND monotherapy increases corneal VEGF-A, VEGF-C, VEGF-D expression and macrophage infiltration into the cornea

**Figure 3:**
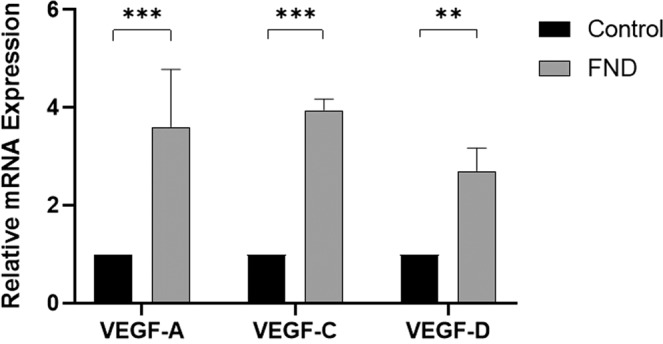
Upregulation of VEGF-A, VEGF-C and VEGF-D after FND monotherapy. Corneas treated with FND were harvested seven days after the treatment and mRNA expression was analysed via qPCR. FND monotherapy induces significant increase of VEGF-A, VEGF-C and VEGF-D expression. (n = 9 in pools of 3, *** = 0.0001 < p ≤ 0.001, ** = 0.001 < p ≤ 0.01).

To further test the hypothesis of a VEGF driven rebound neovascularization effect of FND monotherapy, we investigated the effect of this treatment on the corneal expression of VEGF-A, VEGF-C and VEGF-D. Corneas treated with FND alone were harvested seven days after the treatment and analyzed via qPCR. The expression level of VEGF-A, VEGF-C, VEGF-D was significantly upregulated after FND monotherapy (Fig. 3).

**Figure 4:**
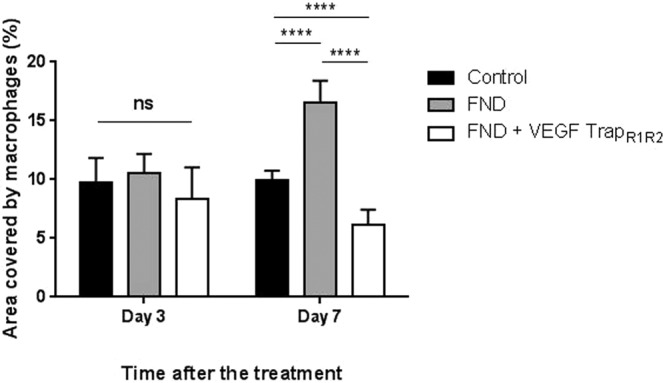
Macrophage infiltration after FND and different pre-treatment. There was no significant difference of LYVE-1 (+) macrophage infiltration between three groups at day 3. However, the infiltration in the FND monotherapy group (16.54 ± 1.87) was significantly increased at day 7 when compared to the control and combined treatment FND + VEGF TrapR_1_R_2_ groups (10 ± 0.76 and 6.17 ± 1.3 respectively). Compared to control group, combined therapy showed significantly less macrophage infiltration into the cornea at day 7. (n = 10; **** = p < 0.0001).

Inflammatory cells, especially macrophages, are important sources of angiogenic and lymphangiogenic growth factors. Therefore, we also studied the infiltration of macrophages into the cornea after different treatments. The area covered by LYVE-1 (+) macrophages was significantly increased in the FND monotherapy group in comparison to the other groups (Fig. 4). In addition, we found an obvious increased expression of monocyte chemoattractant protein-1 (MCP-1) mRNA in the FND treated corneas (Suppl. Figure 1). MCP-1 is responsible for the recruitment of macrophages^29^.

### Macrophage depletion prevents the rebound neovascularization after FND treatment

**Figure 5:**
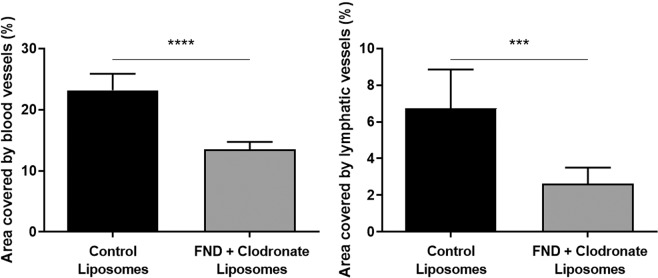
Macrophage depletion prevents the rebound effect of FND monotherapy at day 7. Both blood and lymphatic vessels were significantly decreased in the group of macrophage depletion compared to control group. (n = 10; **** = p < 0.0001, *** = 0.0001 < p ≤ 0.001).

Macrophages are a well known source for VEGFs^30^. To investigate the source of VEGF in the FND model, inducing the rebound of neovascularization, we performed corneal macrophage depletion as previously described^31^. Mice were treated with subconjunctival injections of 10 µl clodronate liposomes one day before FND treatment and every other day afterwards for one week. Control group was treated with subconjunctival injection of equal amounts of control liposomes with similar regime. Our results show that both blood and lymphatic vessels were significantly reduced at day 7 in the macrophage depleted group compared to the control group (Fig. 5).

## Discussion

Three important conclusions can be drawn from this study:

(i) FND itself as a monotherapy is effective in significantly regressing both mature corneal blood and lymphatic vessels at early time points after treatment.
(ii) FND monotherapy significantly increases the expression of VEGF-A, VEGF-C and VEGF-D - the important pro(lymph)angiogenic mediators – as well as macrophage infiltration into the cornea thereby inducing rebound revascularization.
(iii) The rebound effect of FND monotherapy can be prevented by VEGF-A blockade. The combination therapy of FND and VEGF TrapR_1_R_2_ is a potential and effective treatment to regress mature corneal blood and lymphatic vessels and prevent early recurrence.

Corneal neovascularisation, a sight-threatening condition, occurs in a wide variety of corneal pathologies. The regression of CoNV and especially of mature blood and lymphatic vessels is a challenge for ophthalmologists. The fine needle diathermy technique - first described by Pillai *et al*.^19^ - has been clinically used to obliterate mature pathologic corneal blood vessels since then. Based on clinical experience, many authors assumed that FND monotherapy could induce micro trauma causing release of proangiogenic factors and should therefore be combined with anti-inflammatory eye drops or subconjunctival injection of anti-VEGF and with corneal angiography to reduce the undesired effect of FND^19,20,23,24,26^. Indeed, we recently showed that the combination of FND and corticosteroids can regress not only mature blood vessels but also clinically invisible lymphatic vessels and thereby promote subsequent high-risk corneal graft survival^25^. Nevertheless, there was no study so far formally testing whether FND treatment alone induces release of proangiogenic factors or causes rebound neovascularisation.

To our best knowledge, these findings demonstrate for the first time that FND monotherapy (without corticosteroids) significantly upregulates mRNA expression of VEGF-A, VEGF-C and VEGF-D –main pro(lymph)angiogenic factors - as well as the infiltration of macrophages (important source of (lymph)angiogenic growth factors^30^) thereby induces rebound neovascualrization of blood and lymphatic vessels at 7 and 14 days after the treatment compared to control group (Figs. 1–4). Indeed, with the depletion of macrophages by subconjunctival injection of clodronate liposomes, the rebound neovascularization seen at day 7 after FND monotherapy was totally eliminated. Both blood and lymphatic vessels were significantly reduced compard to control group (Fig. 5). These results are in line with the study of Kiesewetter *et al*.^31^.

Monocyte chemoattractant protein-1 (MCP-1), a chemokine released from corneal epithelial cells and binding to chemokine receptor CCR2, subsequently recruits monocytes/macrophages to the cornea in the cauterization model^29^. Oshima *et al*. also indicated that macrophage infiltration was markedly reduced in CCR2 KO mice and MCP-1 KO mice^32^. In fact, our findings showed that FND also causes an upregulation of MCP-1 and thereby resulting in the significant influx of macrophages after the treatment (Supplementary Fig. S1).

It had been previously shown that mild cautery of the cornea without removal of the corneal epithelium fails to evoke a neovascular response whereas cautery with previous epithelial removal can cause an angiogenic response^33^. This is due to the antiangiogenic function of corneal epithelium^33^. In our experiment, although the corneal epithelium was intact, the circular cauterization provoked rebound (lymph)angiogenesis. Conflicting results may be due to the different models studied and the different approach and intensity of FND applied. We here performed a circular perilimbal cauterization on prevascularized corneas instead of mild central cautery on nonvascularized corneas.

VEGF TrapR_1_R_2_ is a fusion protein containing the domain of VEGFR_1_ and VEGFR_2_ fused to the Fc domain of human IgG. Therefore, this trap can bind and block all forms of VEGF-A. Due to this effect, VEGF TrapR_1_R_2_ is an ideal candidate to dampen the upregulation of VEGF-A caused by FND alone. Indeed, this combination treatment could significantly regress blood and lymphatic vessesels at day 7 and day 14 in comparision with the FND monotherapy. These results are compareable to our previous study where FND was combined with corticosteroids^25^. Corticosteroids are clinically used to control inflammation and CoNV due to their anti-inflammatory and anti-angiogenic properties^34^. Many studies recently showed the additional antilymphangiogenic properties of corticosteroids^35–37^. Both dexamethason and anti-VEGF treatments delay cell migration, vessel dilation at the early phase and decrease the affected area of vascularization and the expression of VEGF^33,36,38–41^. However, long-term use of topical corticosteroids can result in severe side effects namely cataract, glaucoma, herpes simplex recurrence and superinfection. The long-term use of anti-VEGF seems to be more safe. However, they were reported to cause epithelial defects, corneal stromal thinning or neurotropic keratopathy. Clinically, Koenig^20^, Elbaz^23^ and Hussain^24^ combined FND with topical or subconjunctival injection of Bevacizumab to treat corneal neovascularisation. This combined treatment was reported to succesfully treat lipid keratopathy and CoNV in preparation of high risk keratoplasty without causing any serious side effects^42^. However, these studies lacked a control group and evidences why FND should be combined with anti-VEGFs^42^.

Taken together, fine needle cautery is an option for treatment of mature pathological corneal neovascularisation. However, as a monotherapy FND results in significant upregulation of VEGF-A, VEGF-C and VEGF-D. VEGF TrapR_1_R_2_ mainly binds VEGF-A and inhibits progressive corneal (lymph)angiogenesis^18^. Our results show that FND combined with VEGF TrapR_1_R_2_ compensates for the limitation of the FND monotherapy and can effectively regress both mature corneal blood and clinically invisible lymphatic vessels in high-risk eyes with reduced risk of recurrence.

## Methods

### Mice and anesthesia

All experimental procedures were approved by Landesamt fuer Natur, Umwelt und Verbraucherschutz Nordrhein-Westfalen, Germany (license: AZ 84-02.04.2016.A055) and conformed to the Association for Research in Vision and Ophthalmology’s Statement for the Use of Animals in Ophthalmology and Vision Research. Female BALB/C mice, purchased from Charles River Laboratories, Sulzfeld, Germany (aged 6–8 weeks) were used in the mouse model of suture-induced corneal inflammation and neovascularization assay. Prior to surgery, a mixture of Ketanest (8 mg/kg) and Rompun (0.1 ml/kg) was intraperitoneally injected to deeply anesthetize the mice.

### Suture-induced corneal inflammation and neovascularisation assay

The mouse model of suture-induced inflammatory neovascularisation was performed as previously described^9,30,43^. Three 11–0 nylon sutures (Serag Wiessner, Naila, Germany) were inserted intrastromally on the right eye with two stromal incursions extending over 120 degrees of corneal circumference each. The outer point of suture placement was chosen near the limbus and the inner suture point was placed near the center of cornea equidistant from the limbus to obtain standardized angiogenic responses. To achieve the peak of (lymph)angiogenesis, sutures were left in place for 14 days and were then removed. After taking out sutures, mice were separated into three groups (n = 10 each): combination treatment of FND and VEGF TrapR_1_R_2_; FND alone and control group complied with the protocols for each group mentioned below.

### Fine needle diathermy and VEGF TrapR_1_R_2_ treatment

FND was performed as previously described under topical anesthesia with Conjuncain®EDO® (Bausch and Lomb, USA)^25^. A stainless steel 3/8 circle round-bodied, single-armed needle was inserted intrastromally close to and parallel to the corneal limbus. Electrode needle connected to diathermy unit with lowest power in soft-coagulating mode was brought into contact with the shaft of the surgical needle. The cauterization was stopped when the vessels were coagulated with corneal blanching and/or corneal shrinkage. This procedure was repeated until reaching 360-degree circumferential cauterization.

After the cauterization, group treated with only FND were not received any further treatment, whereas, combination treatment group was additionally treated with topical VEGF TrapR_1_R_2_ (Eylea 40 mg/ml, Bayer) three drops (3 µl/drop) per day until the end of experiments. Mice in the control group did not obtain FND or VEGF TrapR_1_R_2_.

To investigate the angiogenic stimulus of FND procedure when solely using and the effect of combination treatment (FND + VEGF TrapR_1_R_2_) on regression of both corneal blood and lymphatic vessels, mice were euthanized and corneas were harvested one and two weeks after the treatment. Whole-mount double-immunohistochemistry using CD31 and LYVE-1 was performed as described below.

### Fine needle diathermy on cornea with depletion of macrophages

To investigate the source of VEGF which induces the rebound of neovascularization, mice in treated group received FND therapy and subconjunctival injections of 10 µl clodronate liposomes (Liposoma Research, Amsterdam, Netherlands) to deplete macrophages as previously described^31^. Clodronate liposomes were injected one day before FND treatment and every other day afterwards for the duration of one week. Control group received equal amounts of control liposomes (Liposoma Research, Amsterdam, Netherlands). Seven days after FND treatment, corneas were harvested and stained with CD31 for blood vessels and LYVE-1 for lymphatic vessels.

### Immunohistochemistry and morphological analysis of corneal flat mounts

The blood and lymphatic vessels in corneal wholemounts were double stained as described previously^44–46^. Excised corneas were rinsed in PBS and then fixed in acetone for 20 minutes. After washing in PBS three times, and blocking with 2% bovine serum albumin (BSA) in PBS, the corneas were stained overnight (in dark, at 4 °C) with rabbit anti-mouse LYVE-1 (1:200; AngioBio, Del Mar, CA, USA) for lymphatic vessels and with a FITC-conjugated rat anti-mouse CD31 antibody (1:100; BD Pharmingen, BD Biosciences, USA) for blood vessels. On the next day, a goat-anti-rabbit Cy3-conjugated secondary antibody (1: 100; Dianova) was added to detect LYVE-1. Finally, samples were mounted on slides using fluorescence mounting media (Sigma).

Double stained wholemount images were assembled automatically from nine to 12 images taken at x100 magnification with a fluorescence microscope (BX53, Olympus Optical Co., Hamburg, Germany). Afterwards, the areas covered with blood and lymphatic vessels, and the infiltration of LYVE-1 (+) macrophages at day three and seven were detected with an algorithm established in the image analyzing program Cell^F (Olympus Soft Imaging Solutions GmbH, Münster, Germany), as previously described^43^.

### mRNA Isolation and real-time Polymerase chain reaction (RT-PCR)

Total RNA was isolated from the corneas using the RNeasy Micro Kit (Qiagen, Germany). As only the centre was analysed, corneas were excised without the limbus. Three corneas per group were pooled in one tube to obtain enough RNA. RNase-free DNase Set (Qiagen) was used to eliminate traces of genomic DNA. Subsequently, single strand cDNA (500 ng per group) was synthesized using the First Strand cDNA Synthesis Kit (Fermentas, USA) according to manufacturers’ protocol. PCR reactions (25 µL) contained 50 ng of cDNA, 0.4 mmol/L of each forward and reverse primer, and master mix (SsoFast EvaGreen Supermix; Bio-Rad, Hercules, CA). The primers used for PCR were shown below (Table 1). All reactions were carried out using a CFX96 real-time system C1000 Thermal Cycler (BioRad) and were repeated in triplicate.

**Table 1:** Primer used for Real time PCR.

mRNA	Product size (bp)	Annealing temperature (°C)	Sequence
HPRT (mouse)	163	60–63	F: 5'-GTTGGATACAGGCCAGACTTTGTTG-3' R: 5'-GATTCAACTTGCGCTCATCTTAGGC-3'
VEGF-A (mouse)	184	60	F: 5'-CATGGATGTCTACCAGCGAAG-3' R: 5'-CATGGTGATGTTGCTCTCTGAC-3’
VEGF-C (mouse)	219	60	F: 5'-AGAACGTGTCCAAGAAATCAGC-3' R: 5'-ATGTGGCCTTTTCCAATACG-3’
VEGF-D (mouse)	86	62.4	F: 5'-ATGGCGGCTAGGTGATTCC-3’ R: 5'-CCCTTCCTTTCTGAGTGCTG-3’
MCP-1 (mouse)	312	60	F: 5'- ACCACAGTCCATGCCATCAC -3' R: 5'- TTGAGGTGGTTGTGGAAAAG -3'

### Statistical analysis

Statistical analyses were performed with Prism 6 version 6.07 (GraphPad Software, San Diego, California, USA). Statistical significance between two groups was determined using Student’s t-test or ANOVA for multiple groups. Data reported as mean ± SD. Values with p < 0.05 was considered statistically significant. Graphs were drawn using Prism6.

## Supplementary information

Supplementary information.

## Data Availability

The datasets generated during and analyzed during the current study are available from the corresponding author on reasonable request.
