# Supplementary material for: Supplemental Anti Vegf A-Therapy Prevents Rebound Neovascularisation After Fine Needle Diathermy Treatment to Regress Pathological Corneal (LYMPH)Angiogenesis
Source: Sci Rep. 2020 Mar 3;10:3908. doi: 10.1038/s41598-020-60705-z (PMC7054535; doi:10.1038/s41598-020-60705-z)
Supplement: Supplementary file 1 — Supplementary information. [file 41598_2020_60705_MOESM1_ESM.docx]

**SUPPLEMENTAL ANTI VEGF A-THERAPY PREVENTS REBOUND NEOVASCULARISATION AFTER FINE NEEDLE DIATHERMY TREATMENT TO REGRESS PATHOLOGICAL CORNEAL (LYMPH)ANGIOGENESIS**

Viet Nhat Hung Le^1,2^, Yanhong Hou^1^, Felix Bock^1,3,*^ and Claus Cursiefen^1,3^

1 Department of Ophthalmology, Faculty of Medicine and University Hospital Cologne, University of Cologne, Germany

2 Department of Ophthalmology, Hue College of Medicine and Pharmacy, Hue University, Hue, Vietnam

3 Centre for Molecular Medicine Cologne (CMMC), University of Cologne, Germany


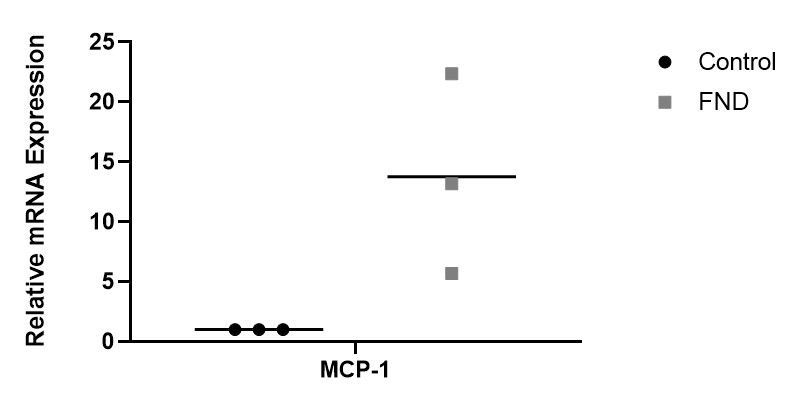


**Supplementary Figure S1:** mRNA expression of MCP-1 after FND monotherapy in the cornea. While MCP-1 is almost absent in control corneas, it is highly, although not significant, upregulated in FND treated corneas. (n = 9 in pools of 3).
